# Crystal structure and Hirshfeld surface analysis of aqua­(1,10-phenanthroline-κ^2^*N*,*N*′)bis­[3-(2-sulf­an­yl­idene-2,3-di­hydro-1,3-benzoxazol-3-yl)propano­ato-κ*O*]zinc(II) monohydrate

**DOI:** 10.1107/S2056989026009722

**Published:** 2026-09-22

**Authors:** Zoxida Rasuljon qizi Mamadaliyeva, Tulqinjon Abdusattor ogli Sattarov, Odil Irgashevich Choriyev, Jalil Tulqin ogli Qadirov, Jamshid Mengnorovich Ashurov

**Affiliations:** a Namangan State University, 161 Boburshoh Street, Namangan 160107, Uzbekistan; b University of Business and Science, 111 Beshkapa Street, Namangan 160107, Uzbekistan; c Institute of Bioorganic Chemistry, Academy of Sciences of Uzbekistan, 100125, M. Ulugbek Str 83, Tashkent, Uzbekistan; d Termez University of Economics and Service, 4-b Farovon Street, Termez 190111, Uzbekistan; University of Neuchâtel, Switzerland

**Keywords:** zinc(II) complex, 1,10-phenanthroline, benzoxazolin-2-thione derivative, crystal structure, Hirshfeld surface analysis, hydrogen bonding, π–π inter­actions

## Abstract

The crystal structure of the title compound, [Zn(C_10_H_8_NO_3_S)_2_(C_12_H_8_N_2_)(H_2_O)]·H_2_O, is reported together with an analysis of its inter­molecular inter­actions. The Zn^II^ ion is five-coordinate, being bonded to two monodentate carboxyl­ate O atoms from two crystallographically independent 3-(2-sulfanylidene-2,3-di­hydro-1,3-benzoxazol-3-yl)propano­ate ligands, two N atoms of a chelating 1,10-phenanthroline ligand and one water O atom. The coordination geometry is distorted square-pyramidal (τ_5_ ≃ 0.30). Although the two carboxyl­ate ligands adopt the same κ*O* coordination mode, they differ in their Zn—O bond lengths and in the conformations of their propano­ate chains. In the crystal, O—H⋯O hydrogen bonds involving the coordinated and solvent water mol­ecules link the complexes into one-dimensional ribbons parallel to [100], reinforced by C—H⋯O and C—H⋯S contacts. Additional consolidation is provided by π–π stacking inter­actions involving the phenanthroline and benzoxazoline-derived aromatic rings. Hirshfeld surface analysis shows that H⋯H (42.0%), H⋯O/O⋯H (19.1%), H⋯S/S⋯H (13.7%) and H⋯C/C⋯H (11.9%) contacts dominate the inter­molecular surface, while C⋯C contacts (5.8%) are consistent with the observed π–π stacking.

## Abstract 2

The crystal structure of a zinc(II) complex containing 3-(2-sulfanylidene-2,3-di­hydro-1,3-benzoxazol-3-yl)propano­ate and 1,10-phenanthroline ligands is described, together with its supra­molecular features and Hirshfeld surface analysis.

## 1. Chemical context

Heterocyclic compounds containing N, O and S atoms are of considerable inter­est because their diverse donor properties make them useful in coordination chemistry and in the design of biologically active compounds (Contreras *et al.*, 2009 ▸). Benzoxazolin-2-one and benzoxazolin-2-thione derivatives form an important class of such compounds, and substitution at the ring N atom provides a convenient means of modifying their physicochemical and biological properties. Derivatives of these heterocycles have been reported to exhibit anti­cancer, analgesic, anti-inflammatory and neuroprotective activities (Prasher *et al.*, 2023 ▸). In particular, sulfur-containing benzoxazole derivatives are of inter­est because replacement of the carbonyl O atom by S increases the polarizability of the heterocycle and may influence both metal-ion inter­actions and inter­molecular contacts. The ability of this sulfur-containing system to inter­act with Zn centres is illustrated by the inhibition of human carbonic anhydrase II by 2-mercaptobenzoxazole, for which coordination of the S atom to the catalytic Zn ion has been demonstrated crystallographically (Bozdag *et al.*, 2020 ▸).

The ligand used in the present study, 3-(2-sulfanylidene-2,3-di­hydro-1,3-benzoxazol-3-yl)propanoic acid, combines a carb­oxy­lic acid group capable of metal coordination with a benzoxazole-2-thione fragment that can participate in supra­molecular inter­actions. The crystal structure of the free acid and those of its mono­ethano­lammonium and ethyl­enedi­ammonium salts have previously been reported (Ashurov *et al.*, 2017*a* ▸). In combination with this ligand, 1,10-phenanthroline provides a rigid chelating *N*,*N*′-donor environment and an extended aromatic surface that may contribute to the consolidation of the coordination unit and to π-related inter­molecular inter­actions (Sammes & Yahioglu, 1994 ▸).

In this work, we report the synthesis, crystal structure and Hirshfeld surface analysis of the title compound (I). The structural analysis focuses on the coordination environment of the Zn^II^ ion and on the inter­molecular inter­actions responsible for the crystal packing.
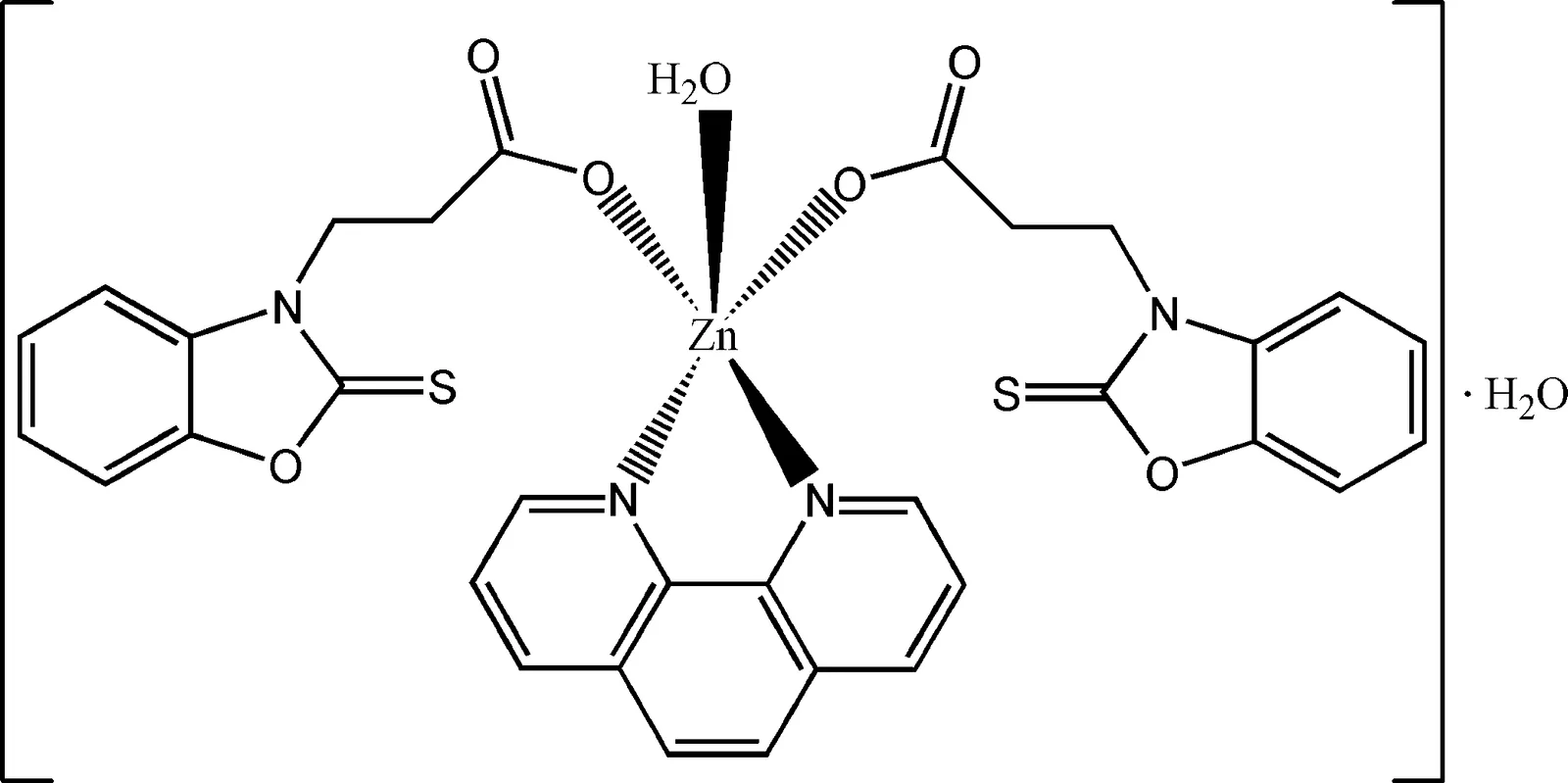


## 2. Structural commentary

The asymmetric unit of the title compound, [Zn(NBA)_2_(phen)(H_2_O)]·H_2_O, where NBA denotes the 3-(2-sulfanylidene-2,3-di­hydro-1,3-benzoxazol-3-yl)propano­ate anion and phen is 1,10-phenanthroline, comprises one neutral Zn^II^ complex mol­ecule and one water mol­ecule of crystallization (Fig. 1 ▸). The two crystallographically independent NBA ligands are distinguished by the suffixes *A* and *B*. The phen ligand is represented by atoms N1, N2 and C1–C12, while O1*W* and O2*W* correspond to the coordinated and solvent water mol­ecules, respectively.

The Zn1 atom is five-coordinate, being bonded to one carboxyl­ate O atom from each of the two independent NBA ligands (O2*A* and O2*B*), the two N atoms of the chelating phen ligand (N1 and N2), and the O atom of the coordinated water mol­ecule O1*W*. The Zn–donor bond lengths are Zn1—O2*A* = 1.9682 (15), Zn1—O2*B* = 2.0457 (17), Zn1—N2 = 2.092 (18), Zn1—O1*W* = 2.133 (2) and Zn1—N1 = 2.1729 (19) Å. Thus, both NBA ligands adopt the same monodentate κ*O* coordination mode, although the Zn1—O2*A* bond is shorter than Zn1—O2*B* by *ca* 0.078 Å.

In both NBA ligands, the second carboxyl­ate O atom lies outside the primary coordination sphere. The Zn1⋯O3*A* and Zn1⋯O3*B* separations are 3.0871 (19) and 2.6375 (18) Å, respectively. The shorter Zn1⋯O3*B* separation may be regarded as a secondary contact, but it is substanti­ally longer than the normal Zn1—O2*B* coordination bond and is not included in the primary coordination number. The corresponding carboxyl­ate C—O distances are O2*A*—C10*A* = 1.282 (3) and O3*A*—C10*A* = 1.224 (3) Å for ligand *A*, and O2*B*—C10*B* = 1.285 (3) and O3*B*—C10*B* = 1.231 (3) Å for ligand *B*.

The phen ligand acts as a conventional chelating κ^2^*N*,*N*′ donor. The Zn1—N1 and Zn1—N2 bond lengths are 2.1729 (19) and 2.0982 (18) Å, respectively, and the N1—Zn1—N2 bite angle is 78.10 (7)°. Coordination generates a five-membered Zn1/N1/C12/C11/N2 chelate ring, while the fused phenanthroline framework remains essentially planar.

The five-coordinate geometry around Zn1 is strongly distorted. The two largest coordination angles are O1*W*—Zn1—N1 = 161.44 (7)° and O2*B*—Zn1—N2 = 143.68 (7)°. These values give an Addison parameter τ_5_ = (β - α)/60 of 0.30, indicating that the coordination geometry is substanti­ally closer to the square-pyramidal limit (τ_5_ = 0) than to the trigonal-bipyramidal limit (τ_5_ = 1). The Zn^II^ centre is therefore best described as having a distorted square-pyramidal five-coordinate environment.

Although the two crystallographically independent NBA ligands have the same κ*O* coordination mode, they differ in both the Zn—O bond lengths and the conformations of their N-substituted propano­ate chains. Their benzoxazoline-2-thione ring systems are essentially planar and the C=S bond lengths are very similar [S1*A*—C7*A* = 1.647 (2) and S1*B*—C7*B* = 1.644 (2) Å]. The conformational differences are reflected in the C1*A*—N1*A*—C8*A*—C9*A* and C1B—N1*B*—C8*B*—C9*B* torsion angles of −92.4 (2) and −112.2 (2)°, respectively, and are particularly pronounced in the N1*A*—C8*A*—C9*A*—C10*A* and N1*B*—C8*B*—C9*B*—C10*B* torsion angles of −179.54 (18) and 61.4 (3)°, respectively. Thus, the two NBA ligands retain the same mode of coordination to Zn^II^ but adopt distinctly different conformations in the crystal.

## 3. Supra­molecular features

The crystal packing is dominated by a cooperative water-mediated O—H⋯O hydrogen-bonding network (Table 1 ▸) involving the coordinated water mol­ecule O1*W*, the water mol­ecule of crystallization O2*W* and the carboxyl­ate O atoms O2*A* and O2*B* (Fig. 2 ▸). The coordinated water mol­ecule acts as a double donor through O1*W*—H1*WA*⋯O2*W*^i^ [H⋯*A* = 1.95 (3) Å, *D*⋯*A* = 2.749 (3) Å and *D*—H⋯*A* = 155 (3)°] and O1*W*—H1*WB*⋯O2*B*^i^ [1.94 (3), 2.751 (3) Å and 159 (3)°, respectively], where symmetry code (i) is −*x*, 1 − *y*, 1 − *z*. The water mol­ecule of crystallization donates through O2*W*—H2*WA*⋯O2*A* [H⋯*A* = 1.96 Å, *D*⋯*A* = 2.797 (3) Å and *D*—H⋯*A* = 170°] and O2*W*—H2*WB*⋯O2*B*^ii^ [2.46, 3.245 (3) Å and 154°, respectively; symmetry code: (ii) 1 − *x*, 1 − *y*, 1 − *z*]. These inter­actions link the complex mol­ecules and water mol­ecules of crystallization into fused cyclic motifs, generating a one-dimensional hydrogen-bonded ribbon parallel to [100] (Figs. 2 ▸ and 3 ▸). The ribbon is reinforced by the nearly linear C1—H1⋯O2*W*^ii^ inter­action [H⋯*A* = 2.39 Å, *D*⋯*A* = 3.319 (4) Å and *D*—H⋯*A* = 178°] and by the inter­molecular C8*A*—H8*AB*⋯S1*A*^iii^ contact [H⋯*A* = 2.87 Å, *D*⋯*A* = 3.716 (2) Å and *D*—H⋯*A* = 146°; symmetry code: (iii) *x* − 1, *y*, *z*], the latter also propagating along [100].

Offset π–π stacking inter­actions provide a further contribution to the crystal packing (Fig. 3 ▸). For the phen ligand, *Cg*5 and *Cg*6 are the centroids of the N1/C1–C4/C12 and N2/C7–C11 rings, respectively. The translational *Cg*5⋯*Cg*6^iv^ inter­action [symmetry code: (iv) 1 + *x*, *y*, *z*] has a centroid–centroid distance of 3.9453 (15) Å, an inter­planar angle of 3.49 (12)° and a slippage of 1.921 Å; the corresponding perpendicular separations are 3.5263 (10) and 3.4460 (11) Å. Ligand A participates in two inversion-related π–π contacts. The *Cg*7⋯*Cg*7^v^ inter­action, where *Cg*7 is the centroid of the C1*A*–C6*A* benzene ring, has a centroid–centroid distance of 3.5541 (15) Å and a slippage of 1.304 Å. A second contact, *Cg*3⋯*Cg*7^v^, occurs at 3.7201 (14) Å with a slippage of 1.722 Å, where *Cg*3 is the centroid of the O1*A*/C6*A*/C1*A*/N1*A*/C7*A* heterocyclic ring. Both contacts involve symmetry code (v), 1 − *x*, 2 − *y*, 2 − *z*. Ligand B displays a different π–π stacking arrangement. The shortest contact is *Cg*4⋯*Cg*8^vi^ = 3.5381 (13) Å, where *Cg*4 and *Cg*8 are the centroids of the O1*B*/C6*B*/C1*B*/N1*B*/C7*B* and C1*B*–C6*B* rings, respectively. This inter­action has an inter­planar angle of 2.41 (12)° and a small slippage of 0.855 Å. A second benzene–benzene inter­action, *Cg*8⋯*Cg*8^vi^, occurs at 3.6998 (14) Å with a slippage of 1.373 Å [symmetry code: (vi) −*x*, 1 − *y*, −*z*]. Thus, ligand *A* exhibits the shorter benzene–benzene stacking contact, whereas the heterocycle–benzene contact involving ligand *B* shows the more direct overlap. Together with the hydrogen-bonding network, these π–π inter­actions consolidate the crystal packing.

## 4. Hirshfeld surface analysis

To qu­antify the inter­molecular contacts contributing to the crystal packing, a Hirshfeld surface analysis (Spackman & Jayatilaka, 2009 ▸) was performed using *CrystalExplorer17* (Turner *et al.*, 2017 ▸). The percentage contributions of the different contact types are summarized in Fig. 4 ▸, while the two-dimensional fingerprint plots and corresponding filtered Hirshfeld surface views are shown in Fig. 5 ▸. H⋯H contacts make the largest contribution, accounting for 42.0% of the total surface. The next most important contributions are H⋯O/O⋯H (19.1%), H⋯S/S⋯H (13.7%) and C⋯C (5.8%) contacts.

The H⋯O/O⋯H contacts are associated with the O—H⋯O and C—H⋯O inter­actions described in the supra­molecular analysis (Table 1 ▸). These short contacts appear as intense red regions on the Hirshfeld surface mapped over *d*_norm_ (Fig. 6 ▸). The appreciable H⋯S/S⋯H contribution is likewise consistent with the observed C—H⋯S inter­actions. Taken together, the H⋯O/O⋯H and H⋯S/S⋯H contacts account for 32.8% of the Hirshfeld surface, emphasizing the importance of hydrogen-bonding and related directional contacts in the crystal packing. C⋯C contacts contribute 5.8% of the surface and are consistent with the π–π stacking inter­actions identified crystallographically, including the phenanthroline and benzoxazoline-derived aromatic stacks discussed above (Figs. 3 ▸ and 7 ▸). Smaller contributions arise from C⋯S/S⋯C (2.7%), O⋯O (1.2%), H⋯N/N⋯H (1.0%), C⋯N/N⋯C (1.0%), O⋯S/S⋯O (0.8%), C⋯O/O⋯C (0.4%) and N⋯S/S⋯N (0.2%) contacts. Overall, the Hirshfeld surface analysis confirms that hydrogen-based contacts dominate the inter­molecular association, while the measurable C⋯C contribution provides additional support for the aromatic π–π stacking that consolidates the crystal structure.

## 5. Database survey

A search of the Cambridge Structural Database (CSD, Version 6.01, November 2025, including the February 2026 updates; Groom *et al.*, 2016 ▸) for the benzoxazole-derived fragment of the ligand used here yielded 215 hits. The most relevant are the free acid YEDCOC and its mono­ethano­lammonium and ethyl­enedi­ammonium salts, YEDCUI and YEDDAP (Ashurov *et al.*, 2017*a* ▸), the related Zn^II^ complex [Zn(*L*)_2_(H_2_O)_4_] (UNODOR; Ashurov *et al.*, 2011 ▸) and its isostructural Co^II^/Cu^II^ analogues (Ashurov *et al.*, 2014 ▸), the (2-oxo-1,3-benzoxazol-3(2*H*)-yl)acetate derivatives NUZSIM (Wang *et al.*, 2016 ▸) with polymorphs NUZSIM01/NUZSIM02 (Ashurov *et al.*, 2017*b* ▸) and the solvate, salts and hydrate ULIBUO, ULICAV, ULICAV01 and ULICOJ (Ashurov *et al.*, 2015*b* ▸), and related tri­ethano­lamine mixed-ligand carboxyl­ate complexes of Zn^II^, Cd^II^ and Cu^II^ (Ashurov *et al.*, 2015*a* ▸, 2016*a* ▸,*b* ▸). No entry corresponding to a Zn^II^ complex of this thione-containing propano­ate ligand together with 1,10-phenanthroline was found.

A further search for the [Zn(phen)(κ*O*-*R*COO)_2_(H_2_O)] fragment – a chelating phen ligand, two monodentate carboxyl­ates and one aqua ligand, as found here – retrieved 61 hits, confirming that this five-coordinate geometry is well established for Zn^II^, even though it has not previously been combined with the present thione-containing ligand. To compare parameters rather than connectivity alone, the Zn—donor bond lengths of the title compound may be set against four representative examples: ADUROH (Luo *et al.*, 2007 ▸), QAHNUK (Liu *et al.*, 2011 ▸), ETUXAT (Nie *et al.*, 2011 ▸) and BUBYOO (Huang *et al.*, 2014 ▸), the last of which, like the title compound, crystallizes with an additional water mol­ecule of crystallization. The Zn1—O(carboxyl­ate) bond lengths in the title compound [1.9682 (15) and 2.0457 (17) Å] compare closely with those in the four analogues [1.988 (3)/2.055 (2), 1.968 (2)/2.000 (2), 2.015 (2)/2.032 (2) and 1.997 (2)/2.064 (2) Å, respectively, in the order above]. Similarly, the Zn1—O(aqua) distance of the title compound [2.133 (2) Å] lies close to the corresponding values [2.030 (3), 2.128 (2), 2.110 (3) and 2.107 (2) Å], and the two Zn1—N(phen) distances [2.098 (2) and 2.173 (2) Å] are comparable with those in the same four structures [2.087 (4)/2.194 (3), 2.103 (2)/2.184 (2), 2.126 (3)/2.131 (2) and 2.095 (2)/2.163 (2) Å]. In every case, including the title compound, the two symmetry-independent Zn—O(carboxyl­ate) bonds are slightly inequivalent [Δ = 0.078 Å in the title compound, *versus* 0.067, 0.032, 0.017 and 0.067 Å in the four analogues, respectively], showing that this small asymmetry is a general feature of bis­(monodentate-carboxyl­ato) coordination at Zn^II^ rather than a peculiarity of the title compound.

## 6. Synthesis and crystallization

Zinc chloride dihydrate, 3-(2-sulfanylidene-2,3-di­hydro-1,3-benzoxazol-3-yl)propanoic acid (HNBA) and 1,10-phenanthroline were mixed in a 1:2:1 molar ratio. An aqueous solution of ZnCl_2_·2H_2_O (0.10 mmol, 17.2 mg) in 5 mL of distilled water was stirred while an ethano­lic solution containing the two organic ligands was added dropwise over approximately 15 min. The ligand solution was prepared by dissolving HNBA (0.20 mmol, 44.7 mg) in 6 mL of warm ethanol, followed by the addition of 1,10-phenanthroline (0.10 mmol, 18.0 mg), dissolved in 4 mL of ethanol. The resulting clear, colourless solution was stirred for a further 30 min at room temperature and then filtered through a fine-porosity glass frit. The filtrate was left to evaporate slowly at room temperature. Colourless single crystals suitable for X-ray diffraction were obtained, collected by filtration, washed with a small amount of cold ethanol–water (1:1, *v*/*v*), and air-dried. The isolated yield was approximately 65% based on Zn. Analysis calculated for C_32_H_28_N_4_O_8_S_2_Zn: C 52.93, H 3.89, N 7.72, S 8.83%. Found: C 52.88, H 3.81, N 7.66, S 8.78%.

## 7. Refinement

Crystal data, data collection and structure refinement details are summarized in Table 2 ▸. H atoms bonded to C atoms were placed in geometrically calculated positions and refined using a riding model, with C—H distances of 0.93 Å for aromatic H atoms and 0.97 Å for methyl­ene H atoms. The H atoms of the coordinated water mol­ecule O1*W* (H1*WA* and H1*WB*) were located from difference-Fourier maps and refined with restrained O—H distances, giving O1*W*—H1*WA* = 0.85 (3) and O1*W*—H1*WB* = 0.86 (3) Å. The H atoms of the water mol­ecule of crystallization O2*W* (H2*WA* and H2*WB*) were retained at fixed positions with O—H = 0.85 Å. Two least-squares restraints were used in the final refinement.

## Supplementary Material

Crystal structure: contains datablock(s) I. DOI: 10.1107/S2056989026009722/tx2116sup1.cif

Structure factors: contains datablock(s) I. DOI: 10.1107/S2056989026009722/tx2116Isup2.hkl

CCDC reference: 2588848

Additional supporting information:  crystallographic information; 3D view; checkCIF report

## Figures and Tables

**Figure 1:**
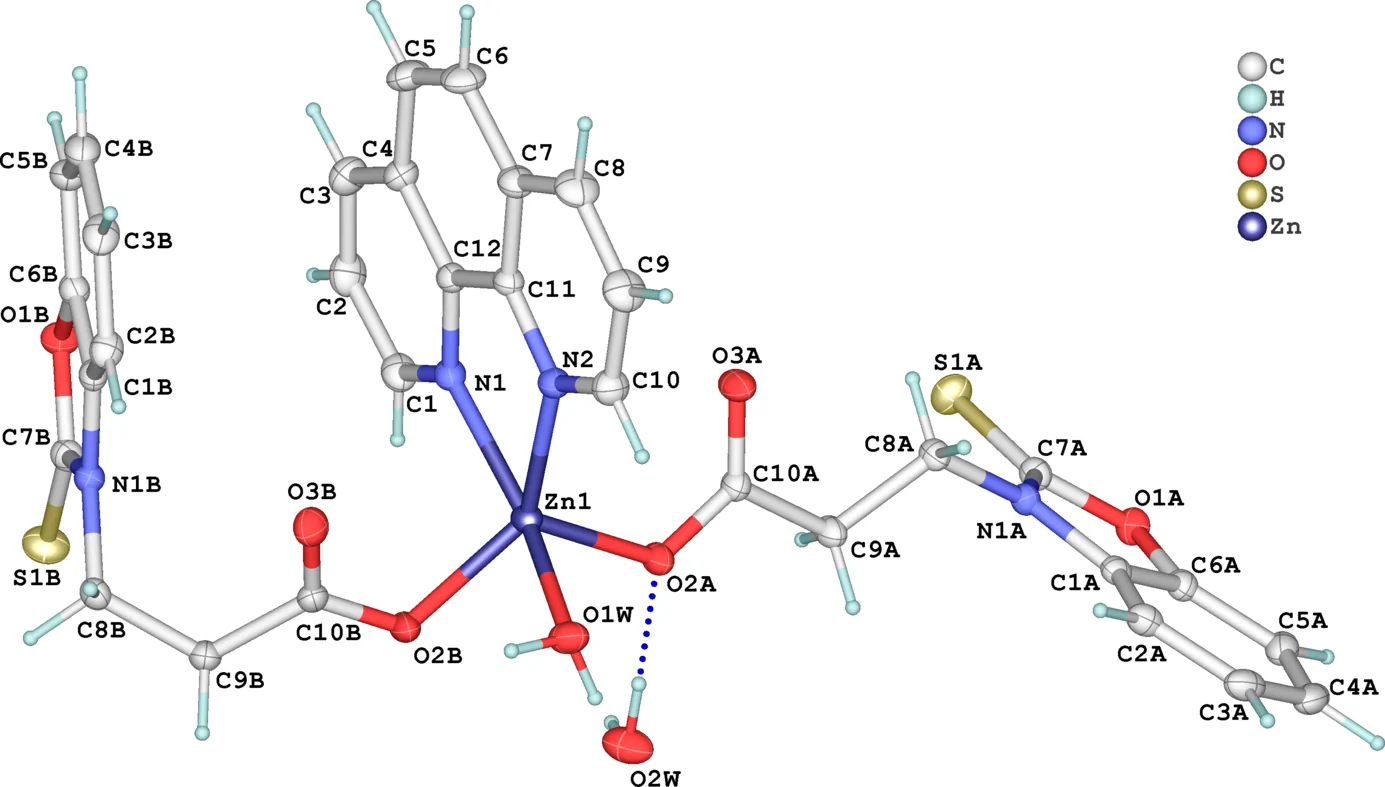
Mol­ecular structure of (I) showing the atom-labelling scheme. Displacement ellipsoids are drawn at the 30% probability level. H atoms are shown as small spheres of arbitrary radii. The dashed line indicates the O—H⋯O hydrogen bond involving the water mol­ecule of crystallization.

**Figure 2:**
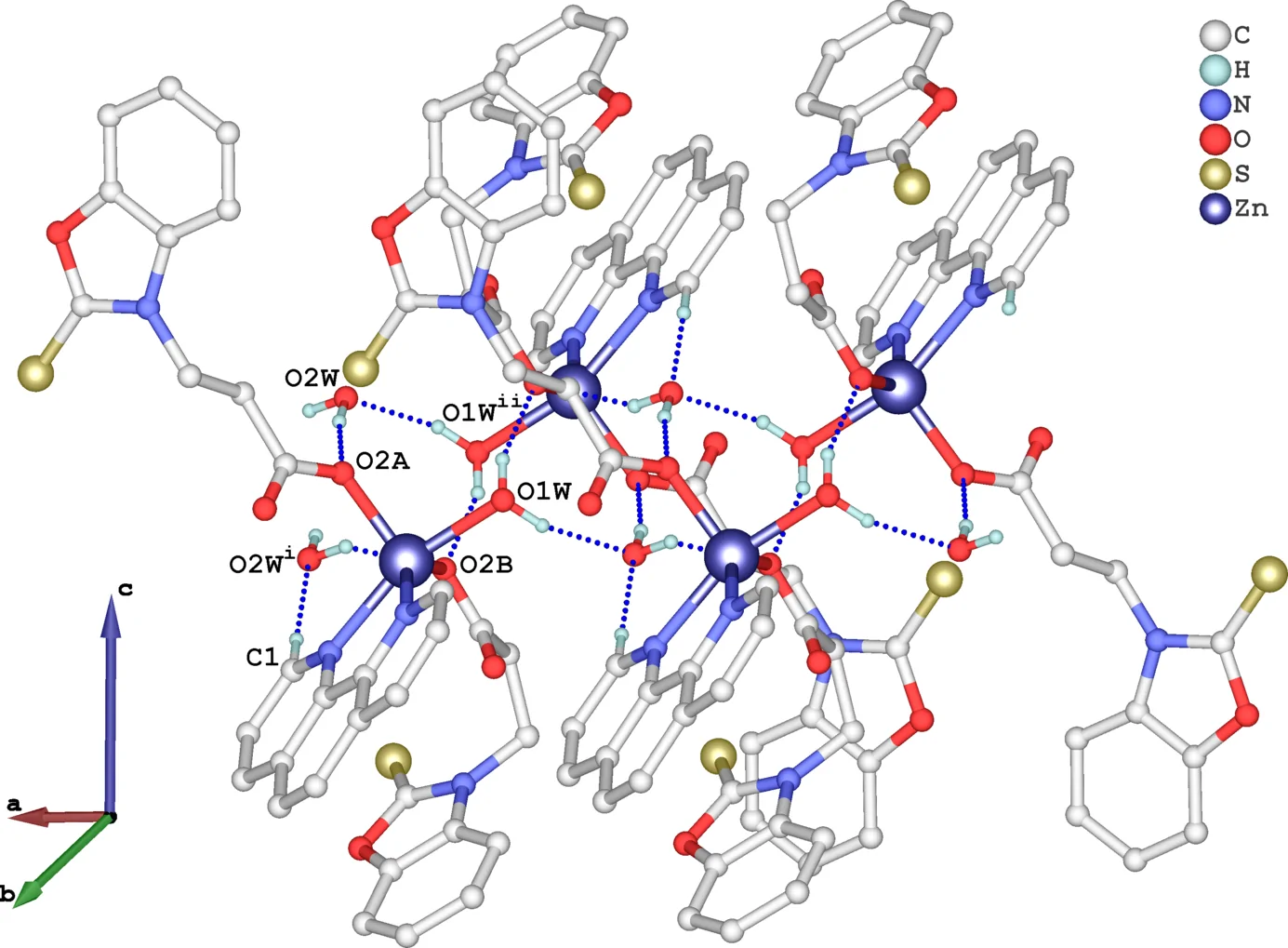
Partial crystal packing showing the inter­molecular O—H⋯O and C—H⋯O hydrogen bonds (blue dashed lines) involving the coordinated and solvent water mol­ecules. H atoms not involved in these inter­actions have been omitted for clarity.

**Figure 3:**
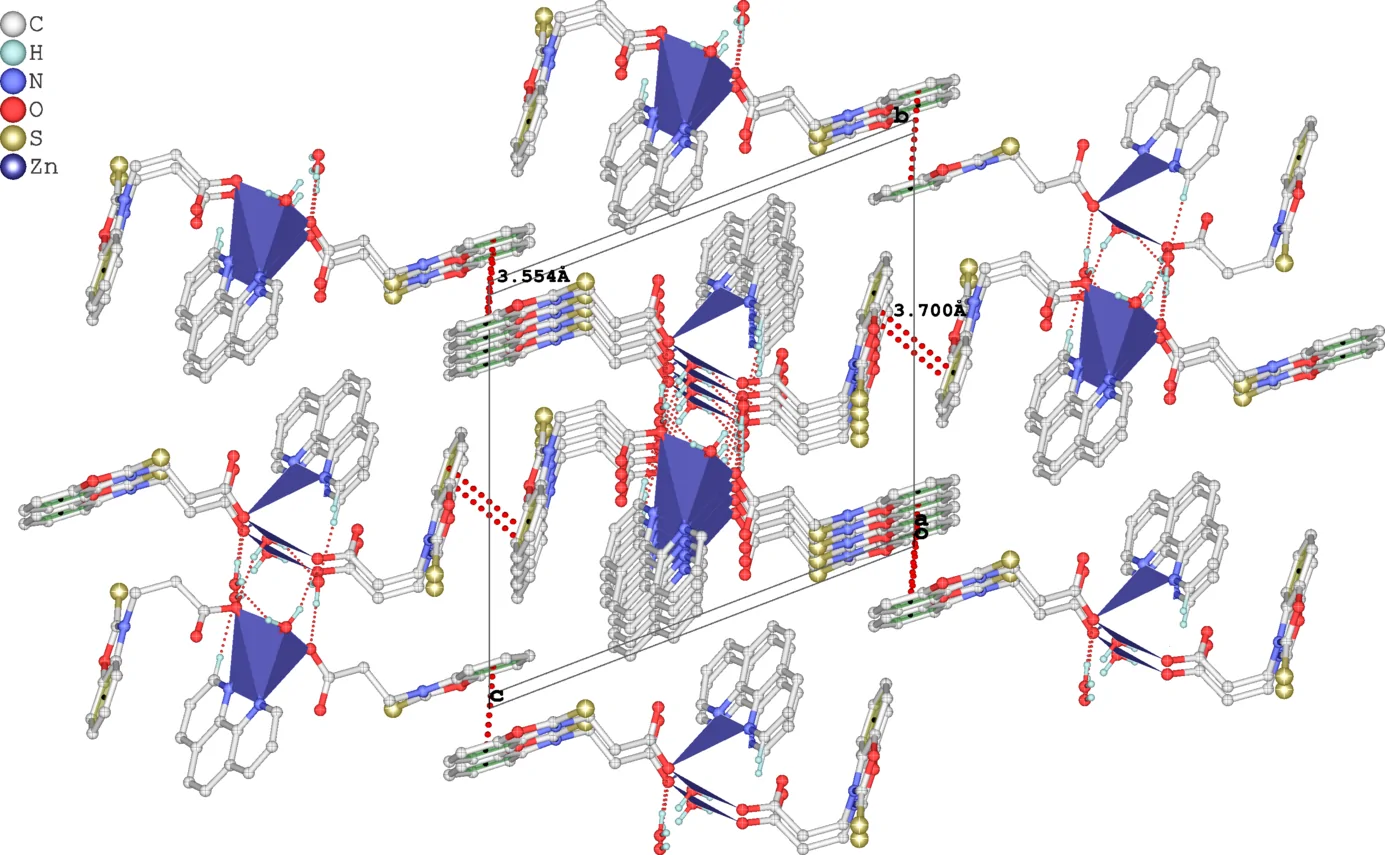
Crystal packing showing the inter­molecular hydrogen-bonding network (cyan dashed lines) and π–π stacking inter­actions (red dotted lines). The centroid–centroid separations for the selected π–π contacts are 3.554 and 3.700 Å. The Zn^II^ coordination polyhedra are shown in blue.

**Figure 4:**
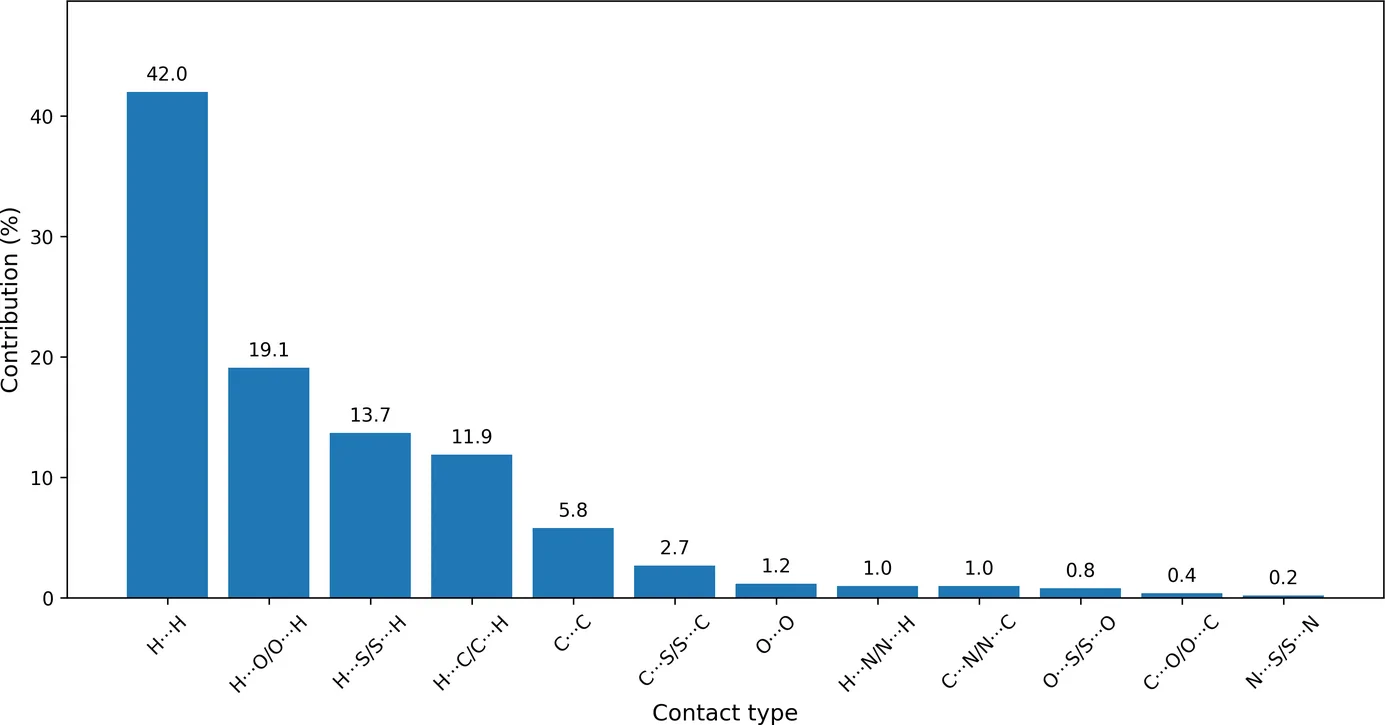
Percentage contributions of the various inter­molecular contacts to the Hirshfeld surface.

**Figure 5:**
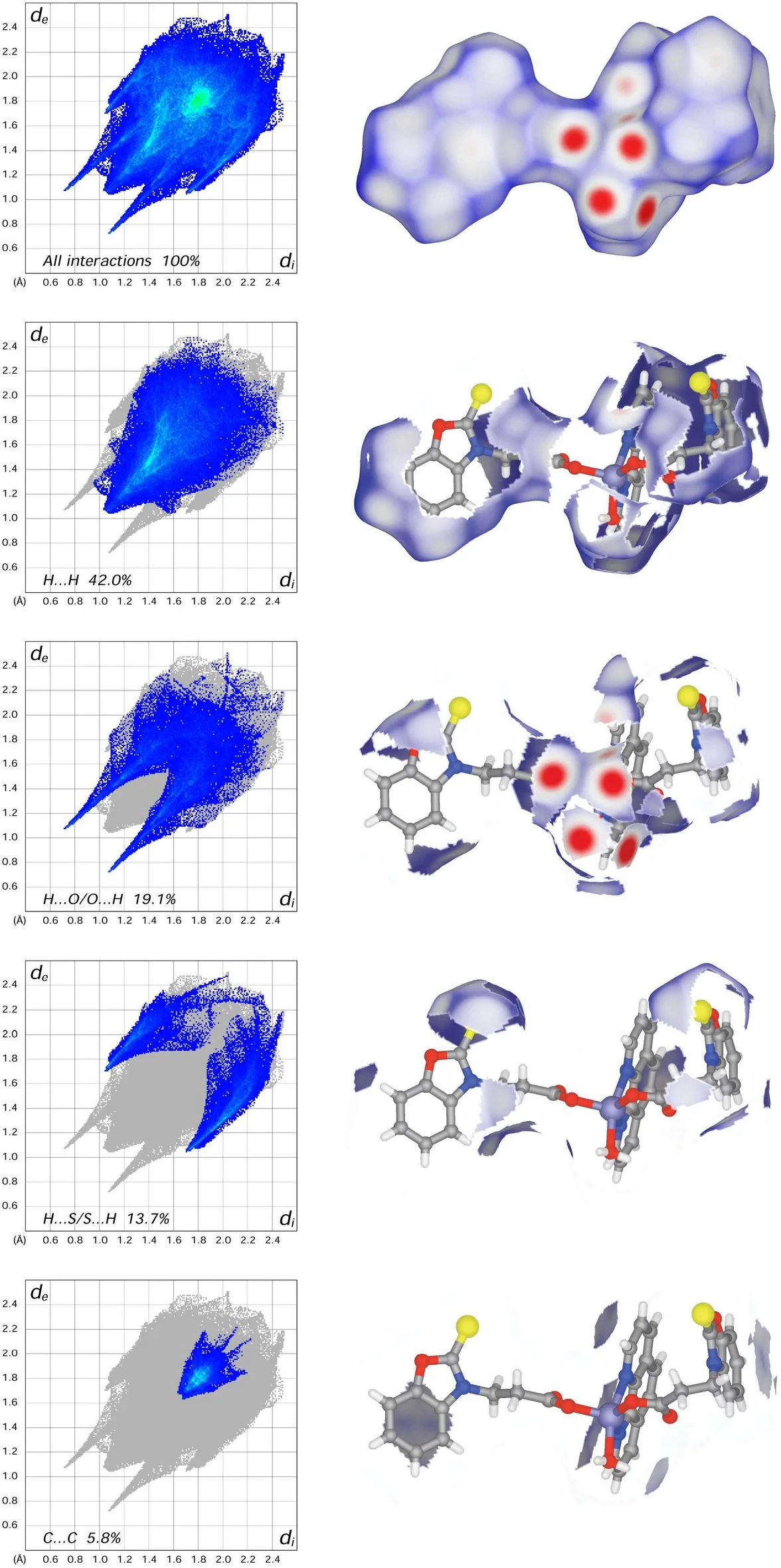
Two-dimensional fingerprint plots (left) and corresponding Hirshfeld surface views (right), showing all inter­molecular contacts and the contributions from H⋯H (42.0%), H⋯O/O⋯H (19.1%), H⋯S/S⋯H (13.7%), and C⋯C (5.8%) contacts.

**Figure 6:**
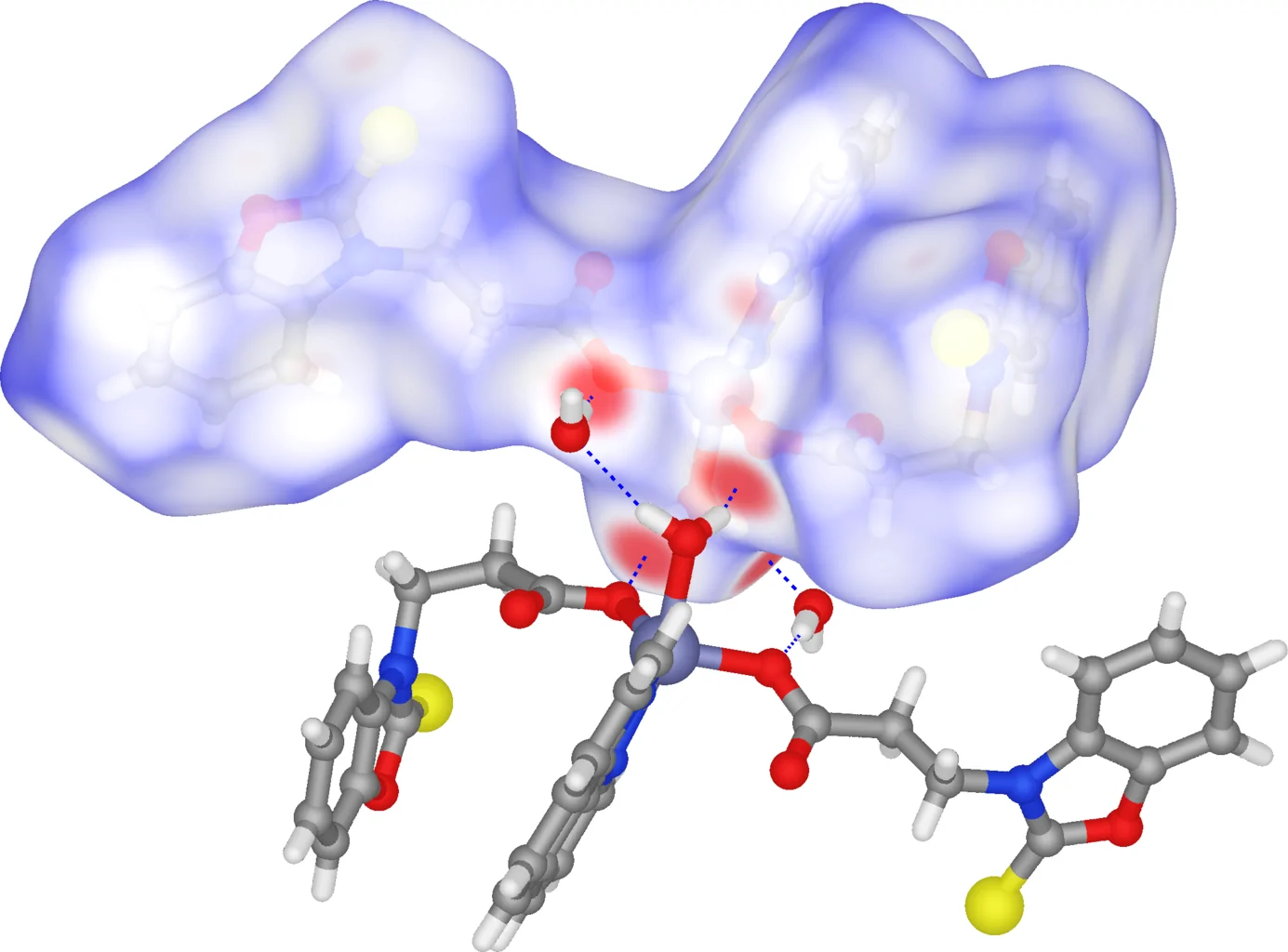
Hirshfeld surface mapped over *d*_norm_ (−0.6163 to 1.3068 a.u.), showing the inter­molecular O—H⋯O hydrogen-bonding contacts.

**Figure 7:**
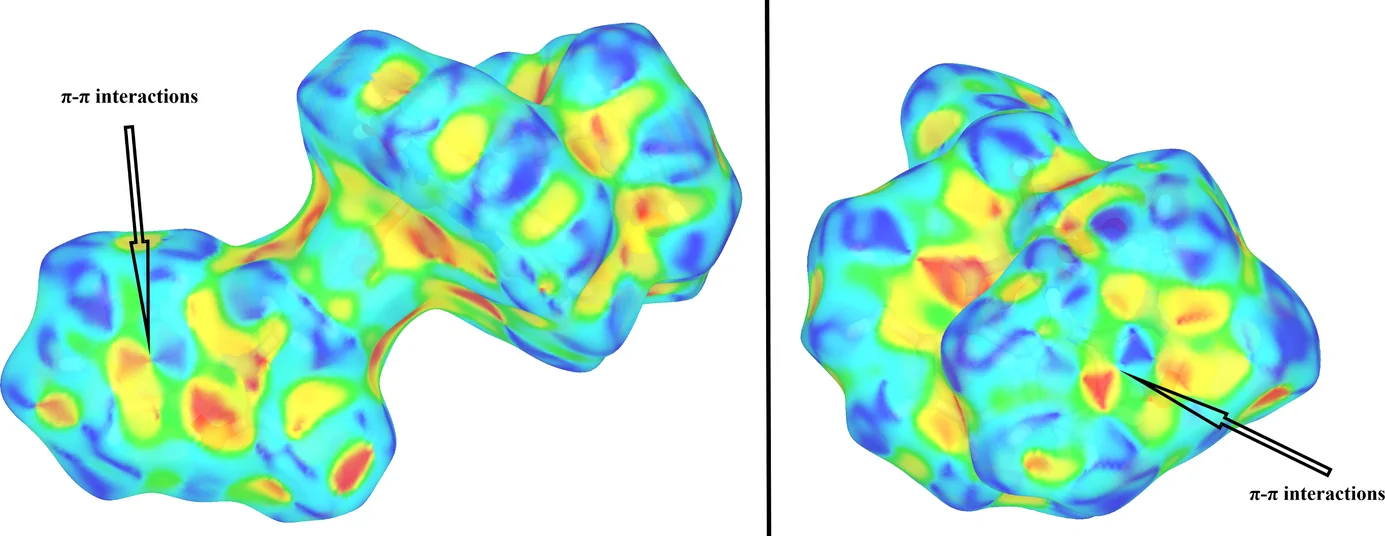
Shape-index surfaces of the two crystallographically independent mol­ecules, *A* (left) and *B* (right), showing the regions involved in π–π stacking inter­actions.

**Table 1:** Hydrogen-bond geometry (Å, °)

*D*—H⋯*A*	*D*—H	H⋯*A*	*D*⋯*A*	*D*—H⋯*A*
O1*W*—H1*WA*⋯O2*W*^i^	0.85 (3)	1.95 (3)	2.749 (3)	155 (3)
O1*W*—H1*WB*⋯O2*B*^i^	0.86 (3)	1.94 (3)	2.751 (3)	159 (3)
O2*W*—H2*WA*⋯O2*A*	0.85	1.96	2.797 (3)	170
O2*W*—H2*WB*⋯O2*B*^ii^	0.85	2.46	3.245 (3)	154
C1—H1⋯O2*W*^ii^	0.93	2.39	3.319 (4)	178
C8*A*—H8*AB*⋯S1*A*^iii^	0.97	2.87	3.716 (2)	146

Symmetry codes: (i) 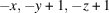; (ii) 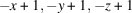; (iii) 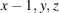.

**Table 2:** Experimental details

Crystal data	
Chemical formula	[Zn(C_10_H_8_NO_3_S)_2_(C_12_H_8_N_2_)(H_2_O)]·H_2_O
*M*_r_	726.07
Crystal system, space group	Triclinic, *P* 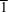
Temperature (K)	291
*a*, *b*, *c* (Å)	6.9071 (3), 14.8406 (7), 16.3048 (7)
α, β, γ (°)	110.602 (4), 94.604 (3), 90.039 (4)
*V* (Å^3^)	1558.65 (13)
*Z*	2
Radiation type	Cu *K*α
μ (mm^−1^)	2.86
Crystal size (mm)	0.3 × 0.22 × 0.14

Data collection	
Diffractometer	Xcalibur, Ruby
Absorption correction	Multi-scan (*CrysAlis PRO*; Agilent, 2014 ▸)
*T*_min_, *T*_max_	0.772, 1.000
No. of measured, independent and observed [*I* > 2σ(*I*)] reflections	11088, 6306, 5486
*R*_int_	0.026
(sin θ/λ)_max_ (Å^−1^)	0.630

Refinement	
*R*[*F*^2^ > 2σ(*F*^2^)], *wR*(*F*^2^), *S*	0.039, 0.109, 1.04
No. of reflections	6306
No. of parameters	436
No. of restraints	2
H-atom treatment	H atoms treated by a mixture of independent and constrained refinement
Δρ_max_, Δρ_min_ (e Å^−3^)	0.34, −0.41

Computer programs: *CrysAlis PRO* (Agilent, 2014 ▸), *SHELXT* (Sheldrick, 2015*a* ▸), *SHELXL2025/1* (Sheldrick, 2015*b* ▸) and *OLEX2* (Dolomanov *et al.*, 2009 ▸).

## supplementary crystallographic information

### Aqua(1,10-phenanthroline-κ2N,N')bis[3-(2-sulfanylidene-2,3-dihydro-1,3-benzoxazol-3-yl)propanoato-κO]zinc(II) monohydrate Crystal data

**Table:**

[Zn(C_10_H_8_NO_3_S)_2_(C_12_H_8_N_2_)(H_2_O)]·H_2_O	*Z* = 2
*M**_r_* = 726.07	*F*(000) = 748
Triclinic, *P*1	*D*_x_ = 1.547 Mg m^−^^3^
*a* = 6.9071 (3) Å	Cu *K*α radiation, λ = 1.54184 Å
*b* = 14.8406 (7) Å	Cell parameters from 6283 reflections
*c* = 16.3048 (7) Å	θ = 5.0–75.8°
α = 110.602 (4)°	µ = 2.86 mm^−^^1^
β = 94.604 (3)°	*T* = 291 K
γ = 90.039 (4)°	Block, colourless
*V* = 1558.65 (13) Å^3^	0.3 × 0.22 × 0.14 mm

### Aqua(1,10-phenanthroline-κ2N,N')bis[3-(2-sulfanylidene-2,3-dihydro-1,3-benzoxazol-3-yl)propanoato-κO]zinc(II) monohydrate Data collection

**Table:**

Xcalibur, Ruby diffractometer	6306 independent reflections
Radiation source: Enhance (Cu) X-ray Source	5486 reflections with *I* > 2σ(*I*)
Graphite monochromator	*R*_int_ = 0.026
Detector resolution: 10.2576 pixels mm^-1^	θ_max_ = 76.3°, θ_min_ = 3.2°
ω scans	*h* = −8→8
Absorption correction: multi-scan (CrysAlisPro; Agilent, 2014)	*k* = −18→17
*T*_min_ = 0.772, *T*_max_ = 1.000	*l* = −16→20
11088 measured reflections	

### Aqua(1,10-phenanthroline-κ2N,N')bis[3-(2-sulfanylidene-2,3-dihydro-1,3-benzoxazol-3-yl)propanoato-κO]zinc(II) monohydrate Refinement

**Table:**

Refinement on *F*^2^	Hydrogen site location: mixed
Least-squares matrix: full	H atoms treated by a mixture of independent and constrained refinement
*R*[*F*^2^ > 2σ(*F*^2^)] = 0.039	*w* = 1/[σ^2^(*F*_o_^2^) + (0.0594*P*)^2^ + 0.2152*P*] where *P* = (*F*_o_^2^ + 2*F*_c_^2^)/3
*wR*(*F*^2^) = 0.109	(Δ/σ)_max_ = 0.002
*S* = 1.04	Δρ_max_ = 0.34 e Å^−^^3^
6306 reflections	Δρ_min_ = −0.41 e Å^−^^3^
436 parameters	Extinction correction: SHELXL-2025/1 (Sheldrick 2015b), Fc^*^=kFc[1+0.001xFc^2^λ^3^/sin(2θ)]^-1/4^
2 restraints	Extinction coefficient: 0.00062 (16)

### Aqua(1,10-phenanthroline-κ2N,N')bis[3-(2-sulfanylidene-2,3-dihydro-1,3-benzoxazol-3-yl)propanoato-κO]zinc(II) monohydrate Special details

**Table:**

Geometry. All esds (except the esd in the dihedral angle between two l.s. planes) are estimated using the full covariance matrix. The cell esds are taken into account individually in the estimation of esds in distances, angles and torsion angles; correlations between esds in cell parameters are only used when they are defined by crystal symmetry. An approximate (isotropic) treatment of cell esds is used for estimating esds involving l.s. planes.

### Aqua(1,10-phenanthroline-κ2N,N')bis[3-(2-sulfanylidene-2,3-dihydro-1,3-benzoxazol-3-yl)propanoato-κO]zinc(II) monohydrate Fractional atomic coordinates and isotropic or equivalent isotropic displacement parameters (Å^2^)

**Table:**

	*x*	*y*	*z*	*U*_iso_*/*U*_eq_	
Zn1	0.06762 (4)	0.65940 (2)	0.47043 (2)	0.04569 (11)	
S1A	0.95185 (9)	0.88039 (6)	0.77354 (4)	0.06117 (17)	
S1B	0.45542 (10)	0.36073 (5)	0.12867 (5)	0.06746 (19)	
O1A	0.8755 (2)	0.88855 (12)	0.93312 (10)	0.0491 (4)	
O1B	0.3695 (2)	0.51840 (12)	0.09501 (11)	0.0511 (4)	
O1W	−0.1818 (3)	0.62099 (14)	0.52147 (13)	0.0633 (5)	
H1WA	−0.268 (4)	0.591 (2)	0.4804 (16)	0.085 (11)*	
H1WB	−0.143 (5)	0.589 (2)	0.5539 (19)	0.084 (11)*	
O2A	0.2398 (2)	0.68238 (11)	0.57848 (10)	0.0523 (4)	
O2B	0.1181 (3)	0.51751 (12)	0.40405 (10)	0.0657 (5)	
O3A	0.2952 (3)	0.83758 (12)	0.60212 (12)	0.0635 (5)	
O3B	−0.0825 (3)	0.55723 (13)	0.31048 (11)	0.0605 (4)	
N1	0.2497 (3)	0.70306 (13)	0.38743 (11)	0.0448 (4)	
N1A	0.6125 (2)	0.87668 (12)	0.84374 (11)	0.0394 (4)	
N1B	0.1228 (2)	0.45948 (12)	0.14092 (11)	0.0404 (4)	
N2	−0.0759 (3)	0.77999 (12)	0.46006 (11)	0.0444 (4)	
C1	0.4102 (4)	0.66383 (18)	0.35236 (16)	0.0570 (6)	
H1	0.460365	0.612170	0.365960	0.068*	
C1A	0.5493 (3)	0.87933 (14)	0.92400 (13)	0.0399 (4)	
C1B	0.0551 (3)	0.53864 (14)	0.12113 (12)	0.0408 (4)	
C2	0.5066 (4)	0.6969 (2)	0.29597 (18)	0.0663 (7)	
H2	0.619772	0.668386	0.273219	0.080*	
C2A	0.3672 (4)	0.87639 (16)	0.95211 (16)	0.0512 (5)	
H2A	0.254143	0.870642	0.915339	0.061*	
C2B	−0.1256 (4)	0.57964 (18)	0.12254 (15)	0.0523 (5)	
H2B	−0.231964	0.556153	0.141242	0.063*	
C3	0.4323 (4)	0.7717 (2)	0.27473 (18)	0.0658 (7)	
H3	0.494006	0.793949	0.236581	0.079*	
C3A	0.3636 (4)	0.88257 (17)	1.03909 (18)	0.0616 (7)	
H3A	0.244233	0.880536	1.061111	0.074*	
C3B	−0.1386 (4)	0.65749 (19)	0.09452 (17)	0.0626 (7)	
H3B	−0.256988	0.687391	0.094711	0.075*	
C4	0.2628 (4)	0.81509 (17)	0.31037 (15)	0.0529 (5)	
C4A	0.5315 (5)	0.89164 (18)	1.09390 (17)	0.0641 (7)	
H4A	0.521670	0.895669	1.151636	0.077*	
C4B	0.0199 (4)	0.69217 (19)	0.06617 (17)	0.0629 (7)	
H4B	0.004782	0.744855	0.048179	0.075*	
C5	0.1755 (5)	0.8952 (2)	0.29361 (19)	0.0687 (7)	
H5	0.230319	0.919649	0.255192	0.082*	
C5A	0.7135 (4)	0.89488 (17)	1.06535 (15)	0.0564 (6)	
H5A	0.826852	0.901560	1.102091	0.068*	
C5B	0.1996 (4)	0.65080 (17)	0.06383 (16)	0.0562 (6)	
H5B	0.305907	0.673314	0.044289	0.067*	
C6	0.0172 (5)	0.9358 (2)	0.33178 (19)	0.0674 (7)	
H6	−0.034647	0.988298	0.320036	0.081*	
C6A	0.7157 (3)	0.88755 (15)	0.97876 (14)	0.0446 (4)	
C6B	0.2099 (3)	0.57408 (15)	0.09244 (14)	0.0441 (4)	
C7	−0.0736 (4)	0.89968 (17)	0.39044 (17)	0.0541 (5)	
C7A	0.8072 (3)	0.88153 (15)	0.84943 (14)	0.0432 (4)	
C7B	0.3108 (3)	0.44668 (16)	0.12318 (14)	0.0463 (5)	
C8	−0.2364 (4)	0.9408 (2)	0.4337 (2)	0.0691 (7)	
H8	−0.291333	0.994427	0.425374	0.083*	
C8A	0.4879 (3)	0.87262 (15)	0.76538 (13)	0.0426 (4)	
H8AA	0.551563	0.907883	0.734519	0.051*	
H8AB	0.366806	0.903599	0.783206	0.051*	
C8B	0.0067 (3)	0.39299 (15)	0.16722 (14)	0.0470 (5)	
H8BA	0.026357	0.327505	0.128808	0.056*	
H8BB	−0.129891	0.406184	0.159830	0.056*	
C9	−0.3137 (4)	0.9012 (2)	0.4883 (2)	0.0700 (7)	
H9	−0.421087	0.928295	0.517837	0.084*	
C9A	0.4445 (3)	0.77031 (15)	0.70392 (14)	0.0489 (5)	
H9AA	0.379984	0.735210	0.734669	0.059*	
H9AB	0.565726	0.739188	0.686507	0.059*	
C9B	0.0584 (4)	0.40123 (16)	0.26168 (14)	0.0535 (6)	
H9BA	−0.018119	0.353486	0.274145	0.064*	
H9BB	0.194228	0.386427	0.268395	0.064*	
C10	−0.2311 (4)	0.82006 (19)	0.49962 (17)	0.0567 (6)	
H10	−0.286786	0.793191	0.536139	0.068*	
C10A	0.3166 (3)	0.76591 (15)	0.62222 (13)	0.0427 (4)	
C10B	0.0249 (4)	0.49929 (16)	0.32831 (14)	0.0503 (5)	
C11	0.0041 (3)	0.81968 (14)	0.40680 (13)	0.0429 (4)	
C12	0.1766 (3)	0.77751 (14)	0.36722 (13)	0.0421 (4)	
O2W	0.4199 (3)	0.52157 (16)	0.59806 (17)	0.0789 (6)	
H2WA	0.378693	0.573354	0.592736	0.118*	
H2WB	0.535657	0.518267	0.583319	0.118*	

### Aqua(1,10-phenanthroline-κ2N,N')bis[3-(2-sulfanylidene-2,3-dihydro-1,3-benzoxazol-3-yl)propanoato-κO]zinc(II) monohydrate Atomic displacement parameters (Å^2^)

**Table:**

	*U*^11^	*U*^22^	*U*^33^	*U*^12^	*U*^13^	*U*^23^
Zn1	0.0585 (2)	0.03806 (16)	0.04177 (17)	−0.00106 (12)	−0.00615 (12)	0.01790 (12)
S1A	0.0439 (3)	0.0827 (4)	0.0627 (4)	−0.0012 (3)	0.0104 (3)	0.0316 (3)
S1B	0.0560 (4)	0.0598 (4)	0.0937 (5)	0.0164 (3)	0.0080 (3)	0.0354 (3)
O1A	0.0407 (8)	0.0570 (9)	0.0473 (8)	0.0028 (7)	−0.0037 (6)	0.0174 (7)
O1B	0.0435 (8)	0.0511 (9)	0.0630 (10)	0.0006 (7)	0.0058 (7)	0.0252 (7)
O1W	0.0723 (12)	0.0603 (11)	0.0643 (11)	−0.0140 (9)	−0.0059 (9)	0.0332 (9)
O2A	0.0672 (10)	0.0419 (8)	0.0450 (8)	−0.0058 (7)	−0.0153 (7)	0.0160 (6)
O2B	0.1151 (16)	0.0402 (8)	0.0407 (8)	−0.0109 (9)	−0.0088 (9)	0.0163 (7)
O3A	0.0870 (13)	0.0469 (9)	0.0585 (10)	−0.0056 (8)	−0.0189 (9)	0.0262 (8)
O3B	0.0701 (11)	0.0571 (10)	0.0522 (9)	0.0103 (8)	0.0114 (8)	0.0153 (8)
N1	0.0525 (10)	0.0400 (9)	0.0419 (9)	0.0061 (8)	−0.0011 (7)	0.0155 (7)
N1A	0.0375 (8)	0.0395 (8)	0.0386 (8)	−0.0007 (7)	0.0001 (6)	0.0113 (7)
N1B	0.0430 (9)	0.0391 (8)	0.0390 (8)	−0.0001 (7)	0.0035 (7)	0.0137 (7)
N2	0.0507 (10)	0.0397 (9)	0.0436 (9)	0.0018 (7)	0.0007 (7)	0.0163 (7)
C1	0.0594 (14)	0.0517 (13)	0.0561 (13)	0.0126 (11)	−0.0004 (11)	0.0156 (11)
C1A	0.0443 (11)	0.0313 (9)	0.0432 (10)	0.0027 (8)	0.0043 (8)	0.0116 (7)
C1B	0.0476 (11)	0.0382 (10)	0.0341 (9)	0.0032 (8)	0.0011 (8)	0.0102 (7)
C2	0.0620 (16)	0.0728 (17)	0.0582 (15)	0.0139 (13)	0.0134 (12)	0.0140 (13)
C2A	0.0502 (12)	0.0420 (11)	0.0612 (13)	0.0005 (9)	0.0118 (10)	0.0166 (10)
C2B	0.0499 (12)	0.0578 (13)	0.0478 (12)	0.0107 (10)	0.0067 (9)	0.0163 (10)
C3	0.0719 (17)	0.0726 (17)	0.0575 (15)	0.0019 (14)	0.0181 (13)	0.0259 (13)
C3A	0.0730 (17)	0.0454 (12)	0.0734 (16)	0.0061 (11)	0.0307 (14)	0.0243 (11)
C3B	0.0699 (17)	0.0588 (14)	0.0575 (14)	0.0258 (13)	0.0041 (12)	0.0186 (11)
C4	0.0629 (14)	0.0521 (13)	0.0473 (12)	0.0000 (11)	0.0058 (10)	0.0219 (10)
C4A	0.100 (2)	0.0467 (13)	0.0529 (13)	0.0105 (13)	0.0224 (14)	0.0238 (11)
C4B	0.089 (2)	0.0474 (12)	0.0538 (14)	0.0122 (13)	0.0014 (13)	0.0205 (11)
C5	0.089 (2)	0.0644 (16)	0.0696 (17)	0.0039 (14)	0.0143 (15)	0.0429 (14)
C5A	0.0795 (17)	0.0446 (12)	0.0459 (12)	0.0100 (11)	0.0001 (11)	0.0181 (9)
C5B	0.0720 (16)	0.0450 (12)	0.0542 (13)	−0.0040 (11)	0.0050 (11)	0.0206 (10)
C6	0.0830 (19)	0.0566 (15)	0.0789 (18)	0.0114 (13)	0.0064 (15)	0.0442 (14)
C6A	0.0503 (12)	0.0382 (10)	0.0438 (11)	0.0027 (8)	0.0020 (9)	0.0131 (8)
C6B	0.0488 (12)	0.0396 (10)	0.0412 (10)	0.0007 (9)	0.0003 (8)	0.0115 (8)
C7	0.0622 (14)	0.0451 (11)	0.0577 (13)	0.0073 (10)	0.0002 (11)	0.0227 (10)
C7A	0.0379 (10)	0.0429 (10)	0.0461 (11)	−0.0007 (8)	−0.0018 (8)	0.0137 (8)
C7B	0.0466 (11)	0.0430 (11)	0.0477 (11)	−0.0013 (9)	0.0004 (9)	0.0151 (9)
C8	0.0709 (17)	0.0552 (15)	0.086 (2)	0.0230 (13)	0.0087 (14)	0.0306 (14)
C8A	0.0422 (11)	0.0401 (10)	0.0435 (10)	0.0009 (8)	−0.0053 (8)	0.0142 (8)
C8B	0.0536 (12)	0.0399 (10)	0.0437 (11)	−0.0082 (9)	0.0029 (9)	0.0101 (8)
C9	0.0642 (16)	0.0640 (16)	0.0813 (19)	0.0228 (13)	0.0178 (14)	0.0226 (14)
C9A	0.0571 (13)	0.0404 (11)	0.0466 (11)	−0.0030 (9)	−0.0104 (9)	0.0153 (9)
C9B	0.0758 (16)	0.0413 (11)	0.0443 (11)	−0.0052 (11)	0.0046 (10)	0.0162 (9)
C10	0.0585 (14)	0.0563 (14)	0.0569 (13)	0.0062 (11)	0.0110 (11)	0.0206 (11)
C10A	0.0474 (11)	0.0418 (10)	0.0380 (10)	0.0009 (8)	−0.0005 (8)	0.0137 (8)
C10B	0.0712 (15)	0.0421 (11)	0.0394 (11)	−0.0094 (10)	0.0085 (10)	0.0157 (9)
C11	0.0517 (12)	0.0355 (9)	0.0415 (10)	0.0013 (8)	−0.0019 (8)	0.0149 (8)
C12	0.0492 (11)	0.0375 (10)	0.0391 (10)	−0.0006 (8)	−0.0021 (8)	0.0143 (8)
O2W	0.0720 (13)	0.0618 (12)	0.1090 (17)	−0.0081 (10)	−0.0172 (12)	0.0429 (12)

### Aqua(1,10-phenanthroline-κ2N,N')bis[3-(2-sulfanylidene-2,3-dihydro-1,3-benzoxazol-3-yl)propanoato-κO]zinc(II) monohydrate Geometric parameters (Å, º)

**Table:**

Zn1—O1W	2.133 (2)	C3A—H3A	0.9300
Zn1—O2A	1.9682 (15)	C3A—C4A	1.382 (4)
Zn1—O2B	2.0457 (17)	C3B—H3B	0.9300
Zn1—N1	2.1729 (19)	C3B—C4B	1.388 (4)
Zn1—N2	2.0982 (18)	C4—C5	1.434 (4)
S1A—C7A	1.647 (2)	C4—C12	1.406 (3)
S1B—C7B	1.644 (2)	C4A—H4A	0.9300
O1A—C6A	1.382 (3)	C4A—C5A	1.381 (4)
O1A—C7A	1.375 (3)	C4B—H4B	0.9300
O1B—C6B	1.385 (3)	C4B—C5B	1.382 (4)
O1B—C7B	1.372 (3)	C5—H5	0.9300
O1W—H1WA	0.854 (10)	C5—C6	1.339 (4)
O1W—H1WB	0.853 (10)	C5A—H5A	0.9300
O2A—C10A	1.282 (3)	C5A—C6A	1.379 (3)
O2B—C10B	1.285 (3)	C5B—H5B	0.9300
O3A—C10A	1.224 (3)	C5B—C6B	1.374 (3)
O3B—C10B	1.231 (3)	C6—H6	0.9300
N1—C1	1.328 (3)	C6—C7	1.433 (4)
N1—C12	1.347 (3)	C7—C8	1.400 (4)
N1A—C1A	1.400 (3)	C7—C11	1.403 (3)
N1A—C7A	1.341 (3)	C8—H8	0.9300
N1A—C8A	1.465 (2)	C8—C9	1.365 (4)
N1B—C1B	1.395 (3)	C8A—H8AA	0.9700
N1B—C7B	1.351 (3)	C8A—H8AB	0.9700
N1B—C8B	1.468 (3)	C8A—C9A	1.510 (3)
N2—C10	1.327 (3)	C8B—H8BA	0.9700
N2—C11	1.358 (3)	C8B—H8BB	0.9700
C1—H1	0.9300	C8B—C9B	1.515 (3)
C1—C2	1.395 (4)	C9—H9	0.9300
C1A—C2A	1.379 (3)	C9—C10	1.397 (4)
C1A—C6A	1.376 (3)	C9A—H9AA	0.9700
C1B—C2B	1.387 (3)	C9A—H9AB	0.9700
C1B—C6B	1.375 (3)	C9A—C10A	1.520 (3)
C2—H2	0.9300	C9B—H9BA	0.9700
C2—C3	1.362 (4)	C9B—H9BB	0.9700
C2A—H2A	0.9300	C9B—C10B	1.510 (3)
C2A—C3A	1.391 (4)	C10—H10	0.9300
C2B—H2B	0.9300	C11—C12	1.438 (3)
C2B—C3B	1.384 (4)	O2W—H2WA	0.8499
C3—H3	0.9300	O2W—H2WB	0.8500
C3—C4	1.404 (4)		
			
O1W—Zn1—N1	161.44 (7)	C6A—C5A—C4A	115.5 (2)
O2A—Zn1—O1W	94.89 (7)	C6A—C5A—H5A	122.2
O2A—Zn1—O2B	100.29 (7)	C4B—C5B—H5B	122.4
O2A—Zn1—N1	102.93 (7)	C6B—C5B—C4B	115.2 (2)
O2A—Zn1—N2	115.84 (7)	C6B—C5B—H5B	122.4
O2B—Zn1—O1W	91.01 (8)	C5—C6—H6	119.5
O2B—Zn1—N1	90.88 (7)	C5—C6—C7	121.0 (2)
O2B—Zn1—N2	143.68 (7)	C7—C6—H6	119.5
N2—Zn1—O1W	89.57 (8)	C1A—C6A—O1A	109.13 (18)
N2—Zn1—N1	78.10 (7)	C1A—C6A—C5A	123.0 (2)
C7A—O1A—C6A	107.32 (16)	C5A—C6A—O1A	127.9 (2)
C7B—O1B—C6B	107.20 (17)	C1B—C6B—O1B	108.95 (18)
Zn1—O1W—H1WA	112 (2)	C5B—C6B—O1B	127.2 (2)
Zn1—O1W—H1WB	107 (2)	C5B—C6B—C1B	123.8 (2)
H1WA—O1W—H1WB	113 (3)	C8—C7—C6	123.3 (2)
C10A—O2A—Zn1	121.33 (14)	C8—C7—C11	117.6 (2)
C10B—O2B—Zn1	104.12 (15)	C11—C7—C6	119.1 (2)
C1—N1—Zn1	129.16 (16)	O1A—C7A—S1A	122.84 (15)
C1—N1—C12	118.5 (2)	N1A—C7A—S1A	128.85 (16)
C12—N1—Zn1	112.20 (14)	N1A—C7A—O1A	108.30 (18)
C1A—N1A—C8A	126.06 (17)	O1B—C7B—S1B	122.14 (17)
C7A—N1A—C1A	109.79 (17)	N1B—C7B—S1B	129.23 (17)
C7A—N1A—C8A	124.12 (18)	N1B—C7B—O1B	108.60 (18)
C1B—N1B—C8B	126.43 (18)	C7—C8—H8	120.3
C7B—N1B—C1B	109.11 (17)	C9—C8—C7	119.3 (2)
C7B—N1B—C8B	124.12 (18)	C9—C8—H8	120.3
C10—N2—Zn1	127.26 (16)	N1A—C8A—H8AA	109.2
C10—N2—C11	118.4 (2)	N1A—C8A—H8AB	109.2
C11—N2—Zn1	114.32 (14)	N1A—C8A—C9A	111.89 (17)
N1—C1—H1	118.6	H8AA—C8A—H8AB	107.9
N1—C1—C2	122.7 (2)	C9A—C8A—H8AA	109.2
C2—C1—H1	118.6	C9A—C8A—H8AB	109.2
C2A—C1A—N1A	132.7 (2)	N1B—C8B—H8BA	109.1
C6A—C1A—N1A	105.45 (18)	N1B—C8B—H8BB	109.1
C6A—C1A—C2A	121.8 (2)	N1B—C8B—C9B	112.67 (18)
C2B—C1B—N1B	133.0 (2)	H8BA—C8B—H8BB	107.8
C6B—C1B—N1B	106.08 (18)	C9B—C8B—H8BA	109.1
C6B—C1B—C2B	120.8 (2)	C9B—C8B—H8BB	109.1
C1—C2—H2	120.5	C8—C9—H9	120.1
C3—C2—C1	119.0 (2)	C8—C9—C10	119.8 (3)
C3—C2—H2	120.5	C10—C9—H9	120.1
C1A—C2A—H2A	122.2	C8A—C9A—H9AA	109.2
C1A—C2A—C3A	115.6 (2)	C8A—C9A—H9AB	109.2
C3A—C2A—H2A	122.2	C8A—C9A—C10A	112.01 (18)
C1B—C2B—H2B	121.9	H9AA—C9A—H9AB	107.9
C3B—C2B—C1B	116.2 (2)	C10A—C9A—H9AA	109.2
C3B—C2B—H2B	121.9	C10A—C9A—H9AB	109.2
C2—C3—H3	120.0	C8B—C9B—H9BA	108.8
C2—C3—C4	120.1 (2)	C8B—C9B—H9BB	108.8
C4—C3—H3	120.0	H9BA—C9B—H9BB	107.6
C2A—C3A—H3A	118.9	C10B—C9B—C8B	114.0 (2)
C4A—C3A—C2A	122.2 (2)	C10B—C9B—H9BA	108.8
C4A—C3A—H3A	118.9	C10B—C9B—H9BB	108.8
C2B—C3B—H3B	119.1	N2—C10—C9	122.2 (2)
C2B—C3B—C4B	121.8 (2)	N2—C10—H10	118.9
C4B—C3B—H3B	119.1	C9—C10—H10	118.9
C3—C4—C5	124.4 (2)	O2A—C10A—C9A	114.07 (18)
C3—C4—C12	117.0 (2)	O3A—C10A—O2A	125.2 (2)
C12—C4—C5	118.7 (2)	O3A—C10A—C9A	120.73 (19)
C3A—C4A—H4A	119.1	O2B—C10B—C9B	114.9 (2)
C5A—C4A—C3A	121.9 (2)	O3B—C10B—O2B	123.0 (2)
C5A—C4A—H4A	119.1	O3B—C10B—C9B	122.1 (2)
C3B—C4B—H4B	119.0	N2—C11—C7	122.6 (2)
C5B—C4B—C3B	122.0 (2)	N2—C11—C12	117.59 (18)
C5B—C4B—H4B	119.0	C7—C11—C12	119.8 (2)
C4—C5—H5	119.1	N1—C12—C4	122.7 (2)
C6—C5—C4	121.8 (2)	N1—C12—C11	117.61 (19)
C6—C5—H5	119.1	C4—C12—C11	119.7 (2)
C4A—C5A—H5A	122.2	H2WA—O2W—H2WB	104.5
			
Zn1—O2A—C10A—O3A	−1.1 (3)	C4B—C5B—C6B—C1B	0.5 (3)
Zn1—O2A—C10A—C9A	179.76 (15)	C5—C4—C12—N1	−179.3 (2)
Zn1—O2B—C10B—O3B	−6.5 (3)	C5—C4—C12—C11	0.1 (3)
Zn1—O2B—C10B—C9B	172.80 (16)	C5—C6—C7—C8	178.4 (3)
Zn1—N1—C1—C2	176.36 (19)	C5—C6—C7—C11	−0.9 (4)
Zn1—N1—C12—C4	−176.40 (17)	C6—C7—C8—C9	179.8 (3)
Zn1—N1—C12—C11	4.1 (2)	C6—C7—C11—N2	−178.6 (2)
Zn1—N2—C10—C9	179.4 (2)	C6—C7—C11—C12	2.2 (4)
Zn1—N2—C11—C7	178.84 (17)	C6A—O1A—C7A—S1A	179.91 (16)
Zn1—N2—C11—C12	−1.9 (2)	C6A—O1A—C7A—N1A	0.2 (2)
N1—C1—C2—C3	−0.9 (4)	C6A—C1A—C2A—C3A	0.2 (3)
N1A—C1A—C2A—C3A	−178.9 (2)	C6B—O1B—C7B—S1B	175.81 (16)
N1A—C1A—C6A—O1A	−0.8 (2)	C6B—O1B—C7B—N1B	−2.3 (2)
N1A—C1A—C6A—C5A	178.3 (2)	C6B—C1B—C2B—C3B	−0.6 (3)
N1A—C8A—C9A—C10A	−179.54 (18)	C7—C8—C9—C10	−0.6 (5)
N1B—C1B—C2B—C3B	−177.5 (2)	C7—C11—C12—N1	177.62 (19)
N1B—C1B—C6B—O1B	−0.4 (2)	C7—C11—C12—C4	−1.8 (3)
N1B—C1B—C6B—C5B	177.8 (2)	C7A—O1A—C6A—C1A	0.4 (2)
N1B—C8B—C9B—C10B	61.4 (3)	C7A—O1A—C6A—C5A	−178.6 (2)
N2—C11—C12—N1	−1.6 (3)	C7A—N1A—C1A—C2A	−179.9 (2)
N2—C11—C12—C4	178.91 (19)	C7A—N1A—C1A—C6A	0.9 (2)
C1—N1—C12—C4	0.2 (3)	C7A—N1A—C8A—C9A	89.8 (2)
C1—N1—C12—C11	−179.2 (2)	C7B—O1B—C6B—C1B	1.7 (2)
C1—C2—C3—C4	0.9 (4)	C7B—O1B—C6B—C5B	−176.5 (2)
C1A—N1A—C7A—S1A	179.61 (17)	C7B—N1B—C1B—C2B	176.1 (2)
C1A—N1A—C7A—O1A	−0.7 (2)	C7B—N1B—C1B—C6B	−1.1 (2)
C1A—N1A—C8A—C9A	−92.4 (2)	C7B—N1B—C8B—C9B	75.1 (3)
C1A—C2A—C3A—C4A	0.4 (3)	C8—C7—C11—N2	2.1 (4)
C1B—N1B—C7B—S1B	−175.82 (17)	C8—C7—C11—C12	−177.1 (2)
C1B—N1B—C7B—O1B	2.1 (2)	C8—C9—C10—N2	1.2 (5)
C1B—N1B—C8B—C9B	−112.2 (2)	C8A—N1A—C1A—C2A	2.1 (3)
C1B—C2B—C3B—C4B	0.4 (4)	C8A—N1A—C1A—C6A	−177.11 (18)
C2—C3—C4—C5	178.7 (3)	C8A—N1A—C7A—S1A	−2.3 (3)
C2—C3—C4—C12	−0.3 (4)	C8A—N1A—C7A—O1A	177.39 (17)
C2A—C1A—C6A—O1A	179.91 (19)	C8A—C9A—C10A—O2A	−164.8 (2)
C2A—C1A—C6A—C5A	−1.0 (3)	C8A—C9A—C10A—O3A	16.0 (3)
C2A—C3A—C4A—C5A	−0.2 (4)	C8B—N1B—C1B—C2B	2.6 (3)
C2B—C1B—C6B—O1B	−178.00 (19)	C8B—N1B—C1B—C6B	−174.67 (18)
C2B—C1B—C6B—C5B	0.2 (3)	C8B—N1B—C7B—S1B	−2.1 (3)
C2B—C3B—C4B—C5B	0.3 (4)	C8B—N1B—C7B—O1B	175.90 (17)
C3—C4—C5—C6	−177.7 (3)	C8B—C9B—C10B—O2B	−160.3 (2)
C3—C4—C12—N1	−0.3 (3)	C8B—C9B—C10B—O3B	19.1 (3)
C3—C4—C12—C11	179.2 (2)	C10—N2—C11—C7	−1.6 (3)
C3A—C4A—C5A—C6A	−0.6 (4)	C10—N2—C11—C12	177.6 (2)
C3B—C4B—C5B—C6B	−0.7 (4)	C11—N2—C10—C9	−0.1 (4)
C4—C5—C6—C7	−0.9 (5)	C11—C7—C8—C9	−0.9 (4)
C4A—C5A—C6A—O1A	−179.9 (2)	C12—N1—C1—C2	0.4 (4)
C4A—C5A—C6A—C1A	1.2 (3)	C12—C4—C5—C6	1.2 (4)
C4B—C5B—C6B—O1B	178.3 (2)		

### Aqua(1,10-phenanthroline-κ2N,N')bis[3-(2-sulfanylidene-2,3-dihydro-1,3-benzoxazol-3-yl)propanoato-κO]zinc(II) monohydrate Hydrogen-bond geometry (Å, º)

**Table:**

*D*—H···*A*	*D*—H	H···*A*	*D*···*A*	*D*—H···*A*
O1*W*—H1*WA*···O2*W*^i^	0.85 (3)	1.95 (3)	2.749 (3)	155 (3)
O1*W*—H1*WB*···O2*B*^i^	0.86 (3)	1.94 (3)	2.751 (3)	159 (3)
O2*W*—H2*WA*···O2*A*	0.85	1.96	2.797 (3)	170
O2*W*—H2*WB*···O2*B*^ii^	0.85	2.46	3.245 (3)	154
C1—H1···O2*W*^ii^	0.93	2.39	3.319 (4)	178
C8*A*—H8*AB*···S1*A*^iii^	0.97	2.87	3.716 (2)	146

Symmetry codes: (i) −*x*, −*y*+1, −*z*+1; (ii) −*x*+1, −*y*+1, −*z*+1; (iii) *x*−1, *y*, *z*.
